# Reliable set-up for in-loop ^11^C-carboxylations using Grignard reactions for the preparation of [*carbonyl*-^11^C]WAY-100635 and [^11^C]-(+)-PHNO^☆^

**DOI:** 10.1016/j.apradiso.2013.07.023

**Published:** 2013-12

**Authors:** Christina Rami-Mark, Johanna Ungersboeck, Daniela Haeusler, Lukas Nics, Cecile Philippe, Markus Mitterhauser, Matthaeus Willeit, Rupert Lanzenberger, Georgios Karanikas, Wolfgang Wadsak

**Affiliations:** a Radiochemistry and Biomarker Development Unit, Department of Biomedical Imaging and Image-guided Therapy, Division of Nuclear Medicine, Medical University of Vienna, A-1090 Vienna, Austria; b Department of Inorganic Chemistry, University of Vienna, A-1090 Vienna, Austria; c Department of Pharmaceutical Technology and Biopharmaceuticals, University of Vienna, A-1090 Vienna, Austria; d Department of Nutritional Sciences, University of Vienna, A-1090 Vienna, Austria; e Department of Psychiatry and Psychotherapy, Medical University of Vienna, A-1090 Vienna, Austria

**Keywords:** Loop method, Grignard reaction, Carbon-11, [^11^C]-(+)-PHNO, [*carbonyl*-^11^C] WAY 100635

## Abstract

Aim of this work was the implementation of a generalized in-loop synthesis for ^11^C-carboxylations and subsequent ^11^C-acylations on the TRACERlab FxC Pro platform. The set-up was tested using [*carbonyl-*^11^C]WAY-100635 and, for the first time, [^11^C]-(+)-PHNO. Its general applicability could be demonstrated and both [*carbonyl*-^11^C]WAY-100635 and [^11^C]-(+)-PHNO were prepared with high reliability and satisfying outcome.

## Highlights

• Generalized method for in-loop ^11^C-carboxylations implemented.
• Grignard reactions successfully tested.
• First in-loop procedure for [^11^C]-(+)PHNO established.
• Satisfactory synthesis outcome for both [*carbonyl*-^11^C]WAY-100635 and [^11^C]-(+)PHNO.
• No distillation for purification of intermediate required.

## 1 Introduction

Radiolabeling of ^11^C-acyl-chlorides and subsequent acylation of suitable precursor compounds are a tool for the production of carbon-11 labeled PET-tracers (Arai et al., 2009; Luthra et al., 1990; Matarrese et al., 2002; McCarron et al., 1996; Zhang et al., 2006). These carbon-11 synthons are prepared using the reaction of [^11^C]CO_2_ ([^11^C]carbon dioxide) and various organo-magnesium halides (so called Grignard reagents (Walborsky, 1990)) for further acylation reactions leading to demanded radiotracer compounds.

One of these compounds is the well-known PET-tracer, [*carbonyl*-^11^C]WAY-100635, which is a highly potent antagonist at serotonin-1A receptor (5HT_1A_) (Andree et al., 2000; Pike et al., 1996). Since the implementation of Grignard reactions is a challenging task for radio-chemists, many set-ups and procedures have been reported, either using a so called “wet” reaction (Hwang et.al., 1999; Krasikova et al., 2009; Pike et al., 1996) or using an in-loop-method (Krasikova et al., 2003; Matarrese et al., 2002; McCarron et al., 1996). Comparing the published data, all procedures lead to a suitable reaction outcome for further use, but specific radioactivities achieved by loop-methods are superior to those in “wet” methods. In-loop reactions for carbon-11-labeled radioligands have been described for both ^11^C-methylations (Gómez et al., 2008; Wilson et al., 2000, 2009; Iwata et al. 2001) and ^11^C-carboxylations using Grignard reactions (Arai et al., 2009; Courtyn et al., 2001; Krasikova et al., 2003; Luthra et al., 1990; Matarrese et al., 2002; McCarron et al., 1996; Zhang et al., 2006). We previously published an in-loop set-up for the routine production of [*carbonyl*-^11^C]WAY-100635 (Wadsak et al., 2007) which is still in use in our facility in various clinical studies dealing with the (patho-)physiological distribution of serotonin 5HT_1A_ receptor, e.g. basic neuroscience investigations (Hahn et al., 2012; Savli et al., 2012; Witte et al., 2009), studies of several psychiatric disorders including major depression (MDD) (Lanzenberger et al., 2012), anxiety disorders (Lanzenberger et al., 2007; Spindelegger et al., 2009) and temporal lobe epilepsy (Assem-Hilger et al., 2010).

Many other pharmacologically interesting compounds for neurological and psychiatric pathologies could also be obtained using such a two-step synthesis route – first a Grignard reaction and a subsequent [^11^C]acylation. Among these compounds, especially, the dopamine D_2/3_ receptor agonist [^11^C]-(+)-PHNO ([^11^C]-(+)-4-propyl-3,4,4a,5,6,10b-hexahydro-2H-naphtho[1,2-b][1,4]oxazin-9-ol hydrochloride) is of interest. It was described as a superior PET-tracer for the fluctuations of synaptic dopamine (Narendran et al., 2006; Searle et al., 2010; Shotbolt et al., 2012; Willeit et al., 2006). The radiosynthesis of this ^11^C-compound was described in a challenging procedure by Wilson et al. (2005) and, most recently, by Plisson et al. (2012). The technical set-up using the proposed methods is very demanding since it includes a distillation step for the purification of the intermediate ^11^C-acylating agent (see Table 1).

**Fig. 1:**
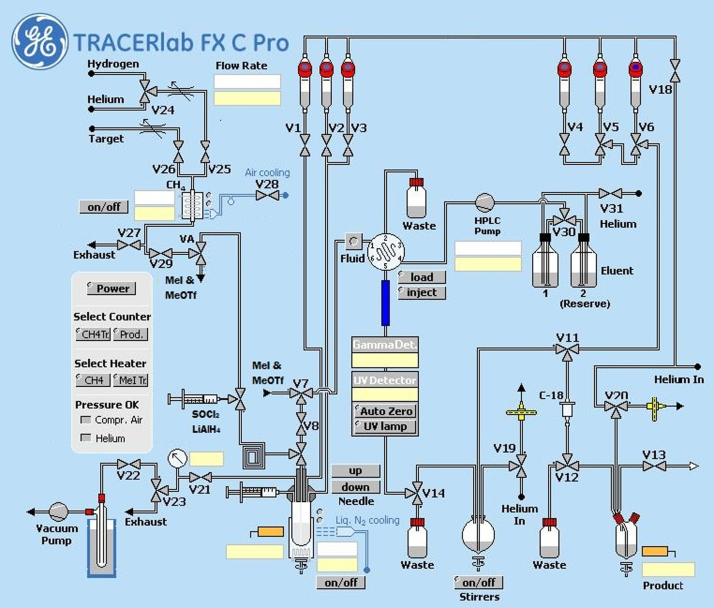
Illustration of synthesizer set-up adapted for in-loop ^11^C-carboxylations.

**Fig. 2:**
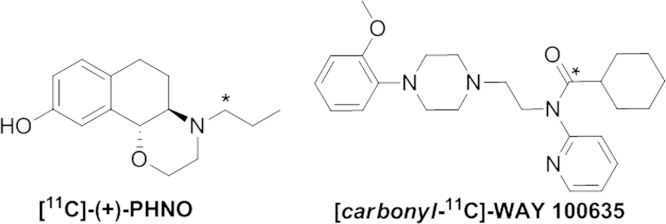
Chemical Structure of [^11^C]-(+)-PHNO and [carbonyl-^11^C]WAY 100635. ^⁎^ indicates the radioactive ^11^C atom.

Since the in-loop method for ^11^C-carboxylation has originally been implemented on site for the preparation of [*carbonyl-*^11^C]WAY-100635 using a Nuclear Interface C11-methylation synthesizer modifications were necessary to translate the set-up to the TRACERlab FxC Pro platform now in use. Moreover, these modifications should enable the implementation of a generalized in-loop synthesis for ^11^C-carboxylations (Fig. 1). Its suitability was tested in the preparation of both [^11^C]-(+)-PHNO and [*carbonyl-*^11^C]WAY-100635 (Fig. 2).

## 2 Materials and methods

### 2.1 General

Grignard reagents were purchased from Sigma Aldrich: ethylmagnesium bromide (3.0 M in diethylether, in Sure-Seal™), cyclohexylmagnesium chloride (2.0 M in diethyl ether) and methylmagnesium bromide (3.0 M in diethylether in Sure-Seal^TM^). Tetrahydrofurane (THF, p.a., without stabilizing agent), thionyl chloride (SOCl_2_, 99%) and triethylamine (TEA, 99.5%) were obtained from Sigma Aldrich (St.Louis, MO, USA). Lithium aluminum hydride (LiAlH_4_, 1 M in THF) was obtained from ABX (Advanced Biochemical Compounds, Radeberg, Germany). 80 cm of a polyethylene (PE) tubing (Fine Bore Polythene Tubing REF 800/100/280; ID: 0.86 mm OD: 1.52 mm, Smiths Medical International Ltd., Kent, UK) was used as “Grignard loop” material. Ascarite II, 20–30 mesh, was obtained from Thomas Scientific (Swedesboro, USA). Sterile water was purchased from Meditrade Medicare Medizinprodukte (Kufstein, Austria). Solid phase extraction (SPE) cartridges (SepPak^®^ C18-plus) were obtained from Waters (Waters^®^ Associates Milford, MA, USA). 0.9% saline solution from B.Braun (Melsungen, Germany), 3% saline solution from a local pharmacy (Landesapotheke Salzburg, Austria), sodium dihydrogenphosphate-monohydrate and disodiumhydrogenphosphate-dihydrate (both from Merck, Darmstadt, Germany) were used for formulation of the product. [^11^C]CO_2_ was produced in a GE PETtrace cyclotron (General Electric Medical System, Uppsala, Sweden) via the ^14^N(p,α)^11^C nuclear reaction by irradiation of a gas target (Aluminum) filled with N_2_ (+1% O_2_) (Air Liquide Austria; irradiation parameters: 16 M eV protons, 10–25 µA h). Preparations were performed on the TRACERlab™ FX C Pro synthesis platform (GE Healthcare, Uppsala, Sweden) including semi-preparative HPLC, SPE purification and product formulation. Chemicals for preparative and analytical High Pressure Liquid Chromatography (HPLC) were obtained from Merck (Darmstadt, Germany) and Sigma-Aldrich (Vienna, Austria) with at least analytical grade and used without further purification. Analytical radio-HPLC runs were performed to verify product identity, to determine radiochemical purity, and to quantify specific activity using a Merck-Hitachi LaChrom system with a UV-detector and NaI-radio detector from Berthold Technologies (Bad Wildbach, Germany).

### 2.2 Radiosyntheses

*In-loop−*^*11*^*C-carboxylation*: 80 cm polyethylene (PE)-tubing was coiled into a loop and equipped with two Luer-fittings. Loops were coated with a mixture of one of the three respective Grignard reagents (500 µL) or diethyl ether (as negative control) in THF (1000 µL) for comparison reasons. Therefore, the respective diluted Grignard solution was pushed through the loop and completely drained by a smooth He-stream (5 mL/min). (*note*: Grignard reagents were purchased as solutions in diethyl ether). The inlet of the loop was connected immediately after impregnation to the line from the molecular sieve CO_2_ trap and the outlet to an Ascarite II trap to collect unreacted [^11^C]CO_2_. Upon delivery from the cyclotron, [^11^C]CO_2_ was transported to the hot cell and trapped on-line within the molecular sieve. Subsequently, it was released by heating the trap to 400 °C and the gas was passed to the previously impregnated loop using a smooth stream of helium (3–5 mL/min). Excess of unreacted [^11^C]CO_2_ (or not combined ^11^C-intermediates) was retained within the Ascarite II trap. The bound ^11^C-acylation synthon was swept out of the loop using a mixture of THF (400 µL) and thionyl chloride (5 µL).

[*Carbonyl-*^*11*^*C*]*WAY-100635*: Precursor compound, WAY-100634, and reference compound, WAY 100635, were obtained from ABX-Advanced Biochemical Compounds (Radeberg, Germany). Semi-preparative HPLC: column: Phenomenex^®^ Gemini, 10 µm 110 A, 250×10 mm^2^; mobile phase: methanol/0.1 M ammonium formate (70/30) (v/v%) plus 3 mL TEA per liter; 8 mL/min; analytical HPLC: column: Waters^®^ µ-Bondapak C-18 (5 µm, 300×3.9 mm^2^ WAT027324) mobile phase: 0.1 M ammonium formate/ACN (55/45 v/v%); 2 mL/min.

Up-scaled [*carbonyl-*^11^C]WAY-100635 production was performed according to Wadsak et al. (2007) with modifications due to the implementation of a new TRACERlab^™^ FX C pro synthesizer. Briefly, loop was coated by pushing a cyclohexane magnesium chloride solution (0.5 mL) in THF (1 mL) through the PE-tubing equipped with Luer-fittings. Radioactivity was trapped nearly quantitatively and converted on-line to magnesium chloride cyclohexane [^11^C]carboxylate. Using a thionyl chloride solution (5 µL in 400 µL THF), the Grignard reaction intermediate was converted to the respective carboxylic acid chloride, swept out the loop and transferred into the reactor vial containing precursor (WAY-100634, 3.4–3.6 mg) in TEA (20 µL) and THF (50 µL). Resulting reaction mixture was heated up to 70 °C for 4 min, cooled down to room temperature and quenched with water (1 mL). Crude reaction mixture was automatically transferred and injected to the semi-preparative HPLC system triggered by a fluid detector. Product peak was collected (6–8 mL) and diluted (80 mL water) within the bulb and passed through an SPE (C-18 plus) column. After complete transfer, the column was washed with water (10 mL) and the purified product was eluted with 1.5 mL ethanol and 5 mL 0.9% saline solution. For final formulation, further 9 mL saline solution 0.9%, 1 mL saline solution 3% and 1 mL phosphate buffer (125 mM) were added, transferred to a lead shielded laminar-air-flow hot cell and sterile-filtered on-line.

[^*11*^*C*]*-*(+)*-PHNO*: Precursor compound, (+)-HNO hydrochloride, and reference compound, (+)-PHNO, were obtained from ABX-Advanced Biochemical Compounds (Radeberg, Germany). Semi-preparative HPLC: column: Phenomenex^®^ Luna C18(2), 10 µm, 250×10 mm^2^; mobile phase: 25 mM PBS (pH 7.0)/acetonitrile (ACN) (60/40 v/v%); 6 mL/min); analytical HPLC: LichroCART^®^ Lichrospher 100, RP-18 (5 µm, 4×250 mm^2^) with LichroCART^®^ Lichrospher RP-18 guard column (5 µm, 4×4 mm^2^), mobile phase: 10 mM PBS (pH 7.0)/ACN (60/40 v/v%); 1.5 mL/min).

**Scheme 1:**
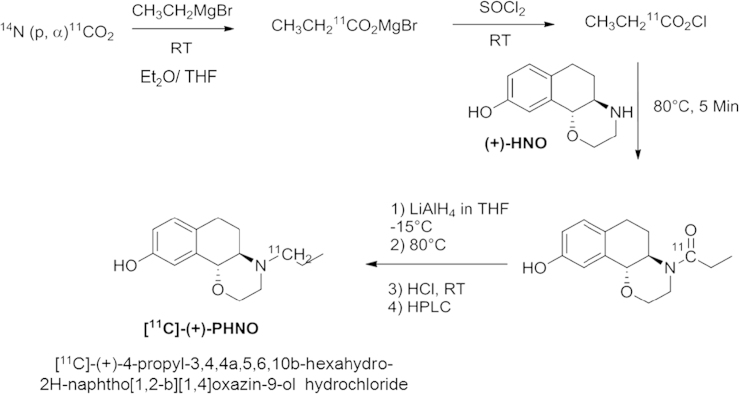
Radiosynthesis of [^11^C]PHNO

The [^11^C]-(+)-PHNO-radiosynthesis is outlined in Scheme 1. The first reaction step in the synthesis sequence, the Grignard reaction, was performed on the basis of the previously described production of [*carbonyl-*^11^C]WAY-100635 adopting the loop method. In this case, the PE-loop was coated with ethyl magnesium bromide (500 µL) in THF (1000 µL) for the conversion to magnesium bromide [^11^C]propionate. Using thionyl chloride solution (5 µL in 400 µL THF), the built [^11^C]propionic acid chloride was transferred directly into the reaction vessel containing (+)-HNO (2.5 mg) suspended in TEA (50 µL) and THF (400 µL). The resulting reaction mixture was heated to 80 °C for 5 min. After cooling down to −15 °C, LiAlH_4_ (120 µL) in THF (400 µL) was added to the reaction intermediate (i.e. [^11^C]1-((4αR,10βR)-9-hydroxy-5,6-dihydro-2H-naphtho[1,2-β][1,4]oxazin-4(3 H,4αH,10βH)-yl)propan-1-one) and subsequently heated up to 80 °C for 2 min. Subsequently, THF was evaporated completely within 2 min and the reaction mixture was cooled to 45 °C. Crude [^11^C]-(+)-PHNO was dissolved in 1 M HCl (800 µL) and neutralized with 1 M sodium hydroxide solution (800 µL) prior to injection onto semi-preparative HPLC. The HPLC fraction containing purified [^11^C]-(+)-PHNO was collected (7–9 mL), diluted with water (70 mL) and passed through a C-18 Sep-Pak^®^ plus cartridge. Further workup procedure and formulation of the final product were identical to the described [*carbonyl*–^11^C]WAY-100635 method (see above).

## 3 Results and discussion

*In-loop−*^*11*^*C-carboxylation*: 3 Grignard reagents were tested regarding the trapping of [^11^C]CO_2_ and release of the respective ^11^C-acylation synthon (^11^C-carboxylic acid chloride). Release and conversion was tested using thionyl chloride in THF for sweeping. All Grignard reagents were able to trap irradiated [^11^C]CO_2_ nearly quantitatively (Table 2). Using a mixture of THF and diethyl-ether alone (i.e. without any Grignard reagent; “negative control”) almost no trapping occurred (2.7±1.1% of initial radioactivity). The release of radioactivity revealed minor differences within the three tested Grignard reagents: [^11^C]propionic acid chloride and cyclohexyl [^11^C]carboxylic acid chloride solution could be transferred to an extent of 98.9±2%. [^11^C]acetic acid chloride solution contained 96.4±1.4% of the trapped radioactivity. Methyl magnesium bromide was chosen as additional Grignard reagent to test whether the described method is able to guarantee reliable outcome for further ^11^C-acylation synthons. Moreover, it might be useful for the development of several radiotracers planned for application in clinical trials in future.

[*Carbonyl−*^*11*^*C*]*WAY-100635*: Overall synthesis outcome for the modified routine-production set-up was 15.5±9.0% (3.4±2.1 GBq, *n*=22) (end of synthesis, EOS). The monitoring of the radioactivity during the production process (Table 3) revealed that irradiated [^11^C]CO_2_ was trapped nearly quantitatively within the impregnated loop and 98±2% (decay corrected) thereof were swept out using thionyl chloride in THF. The routine procedure, including [^11^C]CO_2_ delivery, carboxylation within the coated loop, acylation with precursor compound (WAY-100634), HPLC and SPE purification, formulation and sterile filtration, took 31±3 min in total. The final product was obtained in a total volume of 17.5 mL (containing 8.5% ethanol) as sterile solution which always met the required quality parameters (i.e. pH, osmolality, specific activity, precursor-content, radiochemical and radionuclidic purity, absence of endotoxins, sterility) for human application. Product identity, separation from precursor and specific radioactivity were determined using analytical HPLC. Retention times in the HPLC-analysis were 4.1±0.3 min (*k*′=1.16±0.15) for WAY-100634 and 7.0±0.5 min (*k*′=2.68±0.27) for [*carbonyl−*^11^C]WAY-100635. Due to the implementation of a new synthesizer module (GE TRACERlab^TM^ FX C Pro), and thus a change in the method of [^11^C]CO_2_-trapping, as well as difficulties with the gas supplier at the time of some of the experiments a wide deviation of specific radioactivity (25–348 GBq/µmol) was observed.

**Fig. 3:**
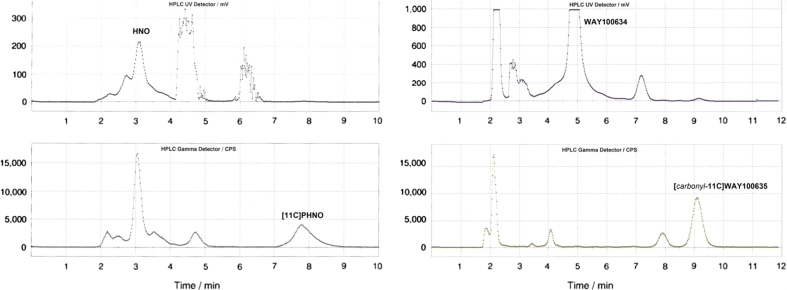
Preparative HPLC Chromatogram of [^11^C]PHNO and [carbonyl-^11^C]WAY100635.

[^*11*^*C*]*-*(*+*)*-PHNO*: In accordance to the experiences with [*carbonyl*-^11^C]WAY-100635, the irradiated [^11^C]CO_2_ was trapped within the ethyl magnesium bromide coated loop nearly quantitatively and could be released by sweeping the loop with thionyl chloride to an extent of 98±2%. During the ^11^C-acylation with the precursor compound, (+)-HNO, about 10% of the radioactivity was lost. Reduction of the intermediate, [^11^C]1-((4aR,10bR)-9-hydroxy-5,6-dihydro-2H-naphtho[1,2-b][1,4]oxazin-4(3H,4aH,10bH)-yl)propan-1-one, with LiAlH_4_ could be obtained in satisfying yields. Upon evaporation of the solvent THF, 59.1±9.6% of the initial radioactivity (at end of bombardement=EOB) were obtained after quenching with hydrochloric acid and neutralization with 1 M NaOH. Radiochemical incorporation yields were measured in the diluted crude mixture and 22.8±10.2% were obtained. Retention times in the preparative HPLC-purification were 3.1±0.2 min (*k*′=0.41±0.09) for (+)-HNO and 7.7±0.4 min (*k*′=2.50±0.18) for [^11^C]-(+)-PHNO (Fig. 3). The product peak showed high pH sensitivity regarding its retention time; therefore neutralization of the crude mixture prior to HPLC purification is mandatory. Furthermore, three unknown (more hydrophilic) radioactive side-products were observed within the preparative chromatogram of the reaction solutions and could be separated from [^11^C]-(+)-PHNO with retention times of 3.05±0.1 min (*k*′=0.39±0.05), 3.55±0.1 min (*k*′=0.61±0.05) and 4.70±0.2 min (*k*′=1.14±0.09). Similar to the results with [*carbonyl*-^11^C]WAY-100635, specific activity was determined via analytical HPLC in a range of 57–305 GBq/µmol (140.6±71 GBq/µmol). Retention times in the respective assay were 2.1±0.1 min (*k*′=0.31±0.06) for (+)-HNO (precursor) and 5.2±0.2 min (*k*′=2.25±0.13) for [^11^C]-(+)-PHNO. Overall synthesis outcome was 8.25±4.2% (1.83±0.55 GBq, range 1.16–3.0 GBq, *n*=11; end of synthesis, EOS) within 37±2 min. Time consumption is in a comparable range to previously published synthesis procedures using the distillation method for the separation of the ^11^C-acylating agent (Plisson et al., 2012; Wilson et al., 2005). Initially, this distillation method was used in a few syntheses but these were stopped since constantly massive contaminations were observed within the hot-cell environment. This was obviously due to the volatility of the transferred compounds and serious problems with leak tightness of the system. Using the in-loop method – and therefore low amounts of immobilized Grignard reagents – it was demonstrated that no separation of excess Grignard intermediate from the crude product was necessary.

## 4 Conclusion

The set-up of a generalized in-loop method allows the reliable ^11^C-carboxylation in a rapid and feasible manner. Its general applicability was demonstrated using three different Grignard compounds in the present work. Modifications and implementation of this procedure on a widely used (commercially available) synthesizer platform was successful and straight forward. Subsequent conversion of the ^11^C-acylating agents without intermediate purification was performed for both [*carbonyl*-^11^C]WAY-100635 and [^11^C]-(+)-PHNO and revealed high reliability and satisfactory outcome. In this work, we present data on the first [^11^C]-(+)-PHNO radiosynthesis comprising an in-loop procedure yielding 8.25±4.2% (1.83±0.55 GBq, range 1.16–3.0 GBq, *n*=11; EOS) within 37±2 min. The presented method might be useful also for future developments and its implementation in other PET centers could help to widen the spectrum of available ^11^C-radiotracers.

## Figures and Tables

**Table 1:** Comparison of reaction parameters for the synthesis of [^11^C]-(+)-PHNO.

Synthesis	Wilson et al. (2005)	Plisson et al. (2012)	Oya et al. (2013)	Present work (2013)
Starting activity ^11^CO_2_ (EOB)	~35 GBq	~70 GBq	~130 GBq	60.7±4 GBq
^11^CO_2_ trapping	Liquid N_2_ cooling	Carbosphere trap	Carbosphere trap	Carbosphere trap
Grignard reaction	In-vial; EtMgBr in diethylether/THF 0.5 M/0.4 mL	In-vial; EtMgBr/THF 250 mM/0.4 mL THF, diethylether	In-vial; EtMgBr in diethylether /THF 0.5 M/0.4 mL	In-loop; EtMgBr/THF (1M/1.5 mL)
Synthesis of acylation reagents	Phthaloyl dichloride in THF (0.5 mL, 2M)+DMF and 2,6-di-tert-butylpyridine; heated to 130 °C and distilled	Phthaloyl dichloride in THF and 2,6-di-tert-butylpyridine; heated to 170 °C and distilled	Phthaloyl dichloride (0.4 mL) and 2,6-di-tert-butylpyridine (0.3 mL), distillation, Ar flow	SOCl_2_/THF (5/400 µL)
Acylation reaction	Trapping −30 °C, 8 µmol HNO 50 µl THF, 50 µL diisopropylethyl-amine, 85°C	Trapped −5 °C, 1–2 mg HNO 0.6 ml THF 50 µL TEA, 80 °C	Trapped −5 °C, HNO in THF Diisopropylethyl-amine, 80 °C	2.5 mg (+)-HNO 400 µL THF, 50 µL TEA, 80 °C
Hydration	−30 °C °C, 0.6 mL 0.2N LiAlH_4_ in THF, 85 °C	−15 °C, 0.1 mL 1M LiALH_4_ in THF 0.2 mL diethylether, 80 °C	0 °C, LiAlH_4_ in THF	−15 °C, 0.52 °mL LiAlH_4_ in THF , 80 °C
Quenching	THF evaporation.1 mL HPLC solvent	THF evaporation 1M HCl	2 M HCl	THF evaporation, 0.8 mL 1M HCL 0.8 mL 1M NaOH
Purification	Semi-preparative HPLC, evaporation and resolubilization	Semi-preparative HPLC, SPE	Semi-preparative HPLC, SPE	Semi-preparative HPLC, SPE
Specific activity(EOS)	33–67 GBq/µmol	84±37 GBq/µmol	18–91 GBq/µmol	140.6±71 GBq/µmol
RCY (EOS)	0.26–0.44 GBq	3.3±1.0 GBq	1.5–4.8 GBq	1.83±0.55 GBq
Synthesis time	40 min	35.4±1.9 min	50–55 min	37±2 min

**Table 2:** In-loop trapping efficiency. 80 cm PE-Loops were coated with a mixture of the respective Grignard reagents (500 µL containing ether)+THF (1000 µL). Calculated percentages of radioactivity are referred to [^11^C]CO_2_ (=100%) at EOB. Values are given as arithmetic means±SD (*n*≥3; corrected for decay).

	Trapping into loop [%]	Release of ^11^C-acylation synthon [%]
THF/ether	2.7±1.1	–
Methyl magnesium bromide	~100	96.4±1.4
Ethyl magnesium bromide	~100	98.9±2
Cyclohexane magnesium chloride	~100	98.9±2

**Table 3:** Progression of radioactivity during [^11^C]-(+)-PHNO synthesis and [carbonyl-^11^C]WAY-100635. Calculated percentages of radioactivity are referred to initially irradiated [^11^C]CO_2_ (=100%). Values are given as arithmetic means±SD (*n*≥8; corrected for decay).

	[^11^C]-(+)-PHNO			[carbonyl-^11^C]WAY 100635		
	Radioactivity (%)	Duration (min)	EOB (min)	Radioactivity (%)	Duration (min)	EOB (min)
Irradiation	100 (60.7±4.4 GBq)	–	–	100 (55.3±8.1 GBq)	–	–
Carboxylation in loop and swept into reactor	98.9±2.0	7	7	98.9±2.0	7	7
Acylation	–	5	13	–	4	12
Reduction with LiAlH_4_	90.0±15.7	6	19	–	–	–
Dilution/addition of HCl	59.1±9.6	4	23	68.8±10.5	2	14
Loop waste/residue in the reactor	15.1±7.0	1	24	11.7±6.0	1	15
Preparative HPLC	–	6	30	–	8	23
SPE, formulation, sterile filtration	–	7	37	–	8	31
Final product	8.2±4.2%	–	37±2	15.5±9.0%	–	31±3
	(1.83 ±0.55 GBq)			(3.4±2.1 GBq)		
